# Antibiofilm activity of a chionodracine‐derived peptide by NMR‐based metabolomics of cell‐free supernatant of *Acinetobacter baumannii* clinical strains

**DOI:** 10.1002/2211-5463.70156

**Published:** 2025-11-02

**Authors:** Fernando Porcelli, Enrico Landi, Francesco Maiurano, Irene Paris, Rosanna Papa, Marco Artini, Laura Selan, Stefano Borocci, Francesco Buonocore, Esther Imperlini

**Affiliations:** ^1^ Department for Innovation in Biological, Agro‐Food and Forest Systems University of Tuscia Viterbo Italy; ^2^ Department of Public Health and Infectious Diseases Sapienza University Rome Italy

**Keywords:** *Acinetobacter baumannii*, antimicrobial peptide, biofilm, extracellular metabolites, metabolic pathways, NMR‐based metabolomics

## Abstract

The ability of *Acinetobacter baumannii* to form biofilm is correlated with its antimicrobial resistance. The identification of antimicrobial drugs acting on biofilm is crucial to develop effective therapies. Previously, we determined that a chionodracine‐derived peptide, KHS‐Cnd, was able to impair *A. baumannii* biofilm formation. Here, to investigate the physiological changes underlying this activity, extracellular metabolite profiles of four *A. baumannii* strains were analyzed by NMR during biofilm formation in the presence of KHS‐Cnd. Metabolites involved in biofilm energy metabolism were found extracellularly after KHS‐Cnd treatment. Significantly altered pathways were associated with glyoxylate/dicarboxylate and branched‐chain/aromatic amino acid metabolism. Overall, differences in extracellular metabolites reflect modifications of biofilm metabolism due to peptide treatment, thus highlighting its therapeutic potential against *A. baumannii* biofilm‐sustained infections.

## Graphical abstract

KHS‐Cnd peptide is able to impair biofilm formation and disaggregate mature biofilms in *Acinetobacter baumannii* clinical isolates. Differences in extracellular metabolites reflect changes in biofilm metabolism due to KHS‐Cnd treatment. Among the differentially represented extracellular metabolites upon KHS‐Cnd treatment, the significantly altered pathways were highly associated with glyoxylate/dicarboxylate and branched‐chain/aromatic amino acid metabolism.

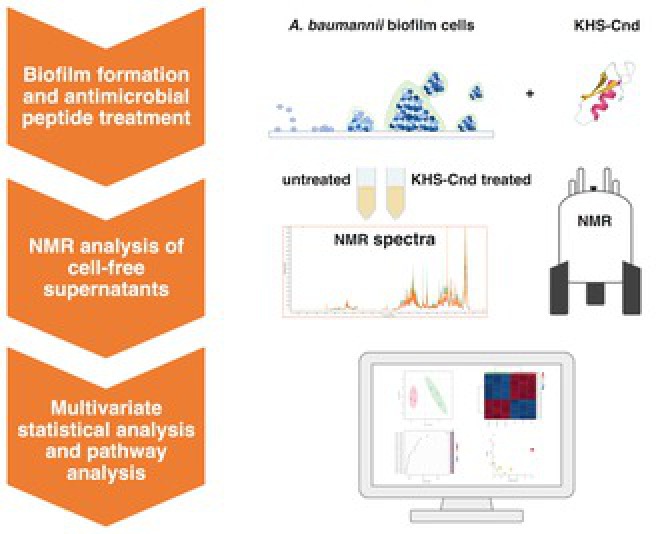

**Abbreviations**
AMP: antimicrobial peptides
AMR: antimicrobial resistance
KHS‐Cnd: KHS‐chionodracine
PCA: principal components analysis
PLS‐DA: partial least‐squares discriminant analysis
VIP: variable importance in projection

*Acinetobacter baumannii* is estimated to be responsible for about 1 million cases of infections per year worldwide, with death rates ranging from 20% to 80% [1]. *A. baumannii* is frequently associated with bloodstream infections and ventilator‐associated pneumonia, where it is responsible for a 35% mortality rate. Moreover, *A. baumannii* community‐acquired pneumonias are associated with a 60% mortality rate [2].

Furthermore, the propensity of *A. baumannii* to adhere and persist on biotic (host mucosal tissue) and abiotic surfaces (e.g., catheters) in biofilm phenotype has contributed to its pathogenicity and drug resistance [3]. Indeed, *A. baumannii* infections are frequently associated with multidrug resistance; recently, some clinical strains have shown resistance to all antibiotics, including colistin and cefiderocol, that are considered last‐resort antibiotics [4].

Biofilm formation is a well‐known and widely described multistep process [5]. The early phase consists of the adhesion of planktonic bacteria to surfaces where pili, flagella, outer membrane proteins, and polysaccharides play a key role in microbial attachment [5]. During this reversible step, a wide range of *A. baumannii* genes are implicated to facilitate initial adhesion to biotic and abiotic surfaces. These genes encode not only surface proteins such as pili and outer membrane proteins but also the chaperone‐usher system that allows bacteria to assemble and secrete pili [6] or the ferric uptake regulator that enables biofilm formation through the control of iron homeostasis [7]. Biofilm formation is also facilitated by outer membrane proteins such as OmpA. OmpA together with biofilm‐associated proteins such as BapAb plays a key role in adherence to host tissues, enhancing biofilm formation and motility, which contribute to *A. baumannii* environmental adaptability and resistance to host defenses [7]. Overall, the features that contribute to the initial bacteria anchoring to surfaces are crucial for the next stages of irreversible attachment and colonization [5]. During the formation and aggregation of microcolonies, these begin to produce all the structural components (polysaccharides, DNA, and proteins) necessary to form extracellular polymeric substances (EPS). During the biofilm maturation stage, in fact, this extracellular matrix provides structural stability to the three‐dimensional biofilm architecture, thus promoting cell‐to‐cell communication [5]. In this context, Quorum Sensing (QS) is a system of cell–cell communication used by bacteria to trigger the expression of genes involved in biofilm formation, virulence factor production, and antibiotic resistance [5, 8]. AbaI/AbaR proteins play as the regulatory axis facilitating adherence of such formed biofilm onto eukaryotic systems and abiotic surfaces with the help of structures such as pili, also under iron‐deficient conditions.

This theory suggests that targeting QS‐related processes in *A. baumannii* could be an effective way to reduce biofilm formation. Various synthetic and natural compounds have been reported to interfere with the QS mechanisms of *A. baumannii*, disrupting biofilm. However, more research is needed to explore the potential of QS inhibition as a treatment strategy [8].

Additionally, bacteriophage‐based strategies have emerged as a promising solution for combating *A. baumannii* biofilms [9].

Therefore, the development of new antimicrobial drugs also able to impair biofilm formation could represent a promising solution to antimicrobial resistance (AMR). In this context, antimicrobial peptides (AMPs) have been considered a valid alternative to conventional antibiotics, exhibiting low predisposition to induce AMR and low toxicity for the host [10, 11, 12]. AMPs can act on several bacterial targets to induce cell death and biofilm destruction, including membrane permeability, intracellular physiological mechanisms, compactness of biofilm extracellular polymeric matrix, cell adhesion, and attachment to the substrate [13, 14, 15]. As an example, a novel peptide, named lariocidin, with broad‐spectrum activity against a range of bacterial pathogens was recently identified and characterized [16]. It has been reported that lariocidin inhibits bacterial growth by binding to the ribosome and interfering with protein synthesis; this mechanism of action was never reported before. We focused our research on studying the biological activity of AMPs mainly identified from Antarctic fishes, an important source of bioactive molecules [11, 17, 18, 19]. The chionodracine, found in the *Chionodraco hamatus*, showed some antibacterial activity against psychrophilic microorganisms, but was not active against human pathogens [20]. Therefore, we designed some chionodracine mutants, starting from the 22 amino acid sequence of this natural molecule, increasing the overall peptide charge and adding a tryptophan residue at the N terminus, and obtaining, in this way, strong antibacterial activity against ESKAPE pathogens [20, 21, 22]. The most promising peptide was the one named KHS‐Cnd in which histidines and serines of chionodracine were all replaced by lysines rising the overall charge from +2 to +7 and maintaining, at the same time, an amphipathic helix conformation [20]. The peptide showed low MIC and MBC concentration values against clinical isolates of *Pseudomonas aeruginosa* (around 1 and 5 μm, respectively) and *A. baumannii* (around 1 and 2 μm, respectively) with low cytotoxicity against a primary human fibroblast cell line and slight hemolytic activity against mammalian erythrocytes at the same time [20]. Successively, we highlighted the antivirulence activity of KHS‐Cnd and its ability to impair biofilm formation and invasion in human pulmonary cells by *P. aeruginosa* [13].

Recently, KHS‐Cnd was tested against other selected reference and clinical *A. baumannii* strains [3], where it showed a significant antibiofilm activity on all tested strains at subinhibitory concentrations during various stages of biofilm formation, including the inhibition of surface‐associated and twitching motilities. Furthermore, the peptide was able to reduce the MIC of ceftazidime/avibactam, when it was used in synergy [3]. These results point out its potential interest as a new antimicrobial agent against *A. baumannii* also in comparison with the smaller activity of other AMPs isolated from frogs [23] or obtained from database screening and *in vitro* modeling [24]. We previously used classical solution NMR methodology to elucidate the 3‐D structures of antimicrobial peptides in the presence of membrane mimicking systems such as micelles of lipids. By the use of NMR, we were able to provide the molecular mechanism of action of AMPs such as pardaxin, magainins, LL‐37, and chionodracine [25, 26].

In this study, we decided to conduct an extracellular metabolic ^1^H NMR analysis on supernatants of bacterial cultures, during *A. baumannii* biofilm formation, treated or not treated with KHS‐Cnd, in order to identify specific metabolites that can be involved in regulating physiological responses of *A. baumannii* during the production of a complex structure that poses significant clinical challenges due to high rates of antibiotic resistance [27, 28].

Metabolite production is fundamental for the proliferation and environmental adaptation of all living organisms. In particular, metabolite change can regulate the physiology and the virulence of pathogenic bacteria during host infection [29]. A recent study has investigated metabolic pathways during antibiotic persistence and pathogenesis of *A. baumannii*, showing that it was able to produce specific derivative metabolites of amino acids and fatty acids [30]. For example, glutamate metabolism is essential to promote resistance to some antibiotics by reducing central carbon metabolism and increasing cell wall construction and stress response. Histidine metabolism, instead, hampers the survival of *A. baumannii* inside macrophages [30]. However, the changes in specific metabolic pathways in response to antibiotic treatments are still poorly explored.

## Materials and methods

### Peptide

The peptide KHS‐Cnd (WFGKLYRGITKVVKKVKGLLKG, overall charge +7, MW 2519.19) was synthesized by Caslo Aps (Caslo Aps Kongens, Lyngby, Denmark) with a purity of 98% (see the Certificate of Analysis, Fig. S1). As previously reported, the peptide concentration was spectrophotometrically determined [31].

### Bacterial strains and growth conditions

Four strains of *Acinetobacter baumannii* were used: the reference ATCC 19606 strain and three clinical strains (Ab1, Ab2, and Ab4) isolated from patients' respiratory infections at the Pediatric Hospital and Institute of Research Bambino Gesù (OPBG) in Rome, Italy. The bacteria were grown in Brain Heart Infusion broth (BHI, Oxoid, Basingstoke, UK) as previously reported [3].

### Biofilm formation and peptide treatment

Bacterial cells, grown in planktonic condition, were diluted 1 : 100 in 24‐well polystyrene flat‐based plates containing medium without or with KHS‐Cnd peptide at a final concentration corresponding to 1/4 of the previously determined minimum inhibitory concentration (MIC) value (1.5 μm for ATCC 19606, Ab1 and Ab4, and 2.5 μm for Ab2, respectively) [3]. The plates were incubated at 37 °C for 18 h under static conditions. After biofilm formation, the KHS‐Cnd treated/untreated bacterial supernatants were recovered from each well, collected by centrifugation at 12 000 rpm for 15 min at 4 °C, filtered through a 0.22 μm filter, and stored at 4 °C until the subsequent analyses.

### ^1^H NMR spectroscopic analysis

NMR samples were obtained from 4 mL of cell‐free bacterial supernatants that were evaporated in a vacuum centrifuge and then dissolved in 600 μL of 0.1 m phosphate buffer pH 7.0, containing 0.3 mm of 3‐trimethylsilyl [2,2,3,3‐d4] propanoic acid (TSP) sodium salt (Sigma, St. Louis, MO, USA) as the internal standard, and 10% (v/v) of D_2_O. Successively, they were transferred into 5 mm NMR tubes. All NMR experiments were performed on three independent replicates for each clinical strain.

### Data acquisition

The 1D ^1^H NMR spectra were acquired on a Bruker Avance III 400 MHz 9.4 T spectrometer (Bruker Biospin, Ettlingen, Germany) equipped with a 5 mm PA BBO probe at 25 ± 1 °C, using the standard Bruker pulse sequence *noesypr1d*, with presaturation during the recycle delay. Data acquisition was carried out using the Topspin software (version 3.5 Bruker Biospin). The *noesypr1D* (Bruker nomenclature) sequence, commonly used in metabolomic studies, was preferred with respect to the classical *1D presat* because of its ability to suppress the peak of water without intensity loss for the majority of peaks, with the exclusion of those very close to the water peak [32]. The *noesypr1D* sequence, commonly used in metabolomic studies, has good water suppression and utilizes the following pulse sequence: RD‐90°‐t1‐90°‐tm‐90°acquire FID: where RD is the relaxation delay of 2 s, and the water is selectively irradiated; t1 and tm were set to 4 μs and 50 ms, respectively. The first pulse creates transverse spin magnetization, the second and the third ones, separated by a mixing time tm, correspond to the NOESY filter, which improves the water suppression [33]. The 90° pulse length was determined by measuring a null spectrum with an approximate 360° pulse and was set to 14.32 μs. Tuning, matching, and shimming for optimal signal and water suppression were performed for each sample. The spectral width was 6410 Hz, and each spectrum was recorded using 128 transients and 32 k points. Free induction decays (FID) were zero filled to 64 k data points and multiplied by an exponential function equivalent to 0.3 Hz line broadening before Fourier transformation. The transformed spectra were referenced to TSP resonance at 0.0 ppm and manually corrected for phase and baseline. Then, the spectra were reduced into spectral bins of 0.02 ppm using the ACD intelligent bucketing method (1D NMR Manager software ACD/Labs, Toronto, Canada), excluding the residual water region δ 4.54–5.22 and the TSP peak δ−0.5–0.5. The ^1^H‐^1^H total correlation spectroscopy (TOCSY) 2D experiments were acquired using the MLEV‐17 spin lock sequence [34] with a spectral width of 10 ppm in both dimensions and a mixing time of 70 ms.

### Spectral data processing and multivariate statistical analysis

NMR spectra of supernatant samples were represented by 408 bins corresponding to a range of chemical shifts in ppm, integrated and transformed into a comma‐delineated form (CSV) for statistical analysis with the Metaboanalyst 6.0 software (http://www.metaboanalyst.ca). The data were standardized using normalization by sum, square root transformation, and Pareto scaling. Principal Components Analysis (PCA) was carried out to perform data dimension reduction and highlight the main variations or similarities among samples. Partial Least Squares Discriminant Analysis (PLS‐DA) was applied to maximize the separation between the groups and to obtain a better prediction of the variables. The most relevant ^1^H NMR variables sensitive to the peptide treatment were identified using the VIP (Variable Importance in Projection) scores with VIP > 1 and *P* < 0.05.

### NMR peak assignment

The assignment of peaks of 1D ^1^H NMR spectra to metabolites was carried out by using Chenomx NMR Suite 10.0, by querying the in‐house NMR database and/or Biological Magnetic Resonance Bank‐ BMRB (https://bmrb.io/metabolomics/) [35, 36]. Unambiguous assignments have been verified and confirmed through 2D ^1^H/^1^H TOCSY spectroscopy. A portion of the TOCSY spectrum is reported in Fig. S2.

### Pathway analysis of NMR data

The significantly represented metabolites were subjected to pathway analysis through Metaboanalyst 6.0 software. *A. baumannii* ATCC 17978 Kyoto Encyclopedia of Genes and Genomes (KEGG) database was queried to find enriched pathways. The enrichment of metabolites in the annotated pathways was assessed by degree centrality and Fisher's exact test. If *P* is ≤ 0.05, a metabolite is considered significantly enriched within the considered pathway network.

## Results

### Effect of KHS‐Cnd peptide on extracellular metabolite profiles of biofilm cells from *Acinetobacter baumannii* clinical isolates

To evaluate changes induced by KHS‐Cnd on cells forming biofilm from *Acinetobacter baumannii* clinical isolates (Ab1, Ab2, and Ab4) and reference strain (ATCC 19606), we analyzed the extracellular metabolite profiles of sessile cultures upon exposure to the antimicrobial peptide by 1D ^1^H NMR analysis. The supernatant samples were collected, after overnight incubation under static conditions, from bacterial cultures treated with sub‐MIC concentrations of KHS‐Cnd, at which the peptide showed significant biofilm activity against all tested *A. baumannii* strains as previously reported [3]. The cell‐free supernatants from KHS‐Cnd treated and untreated sessile cultures of all four strains were then subjected to 1D ^1^H NMR analysis.

### Multivariate statistical analysis of cell‐free supernatants from *Acinetobacter baumannii* sessile cultures treated with KHS‐Cnd peptide

The obtained matrix data from 1D ^1^H NMR was subjected to a pre‐assignment multivariate statistical analysis to highlight the samples' clustering patterns.

**Fig. 1:**
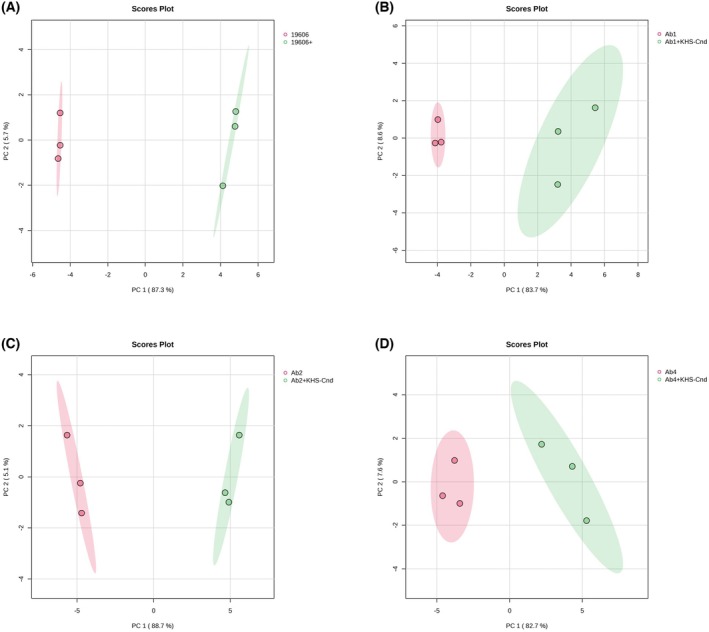
Multivariate statistical analysis applied on the ^1^H NMR data matrix of *A. baumannii* supernatants after KHS‐Cnd treatment during biofilm formation. PCA score plot of cell‐free supernatants from untreated and KHS‐Cnd‐treated sessile cultures of ATCC 19606 (A), Ab1 (B), Ab2 (C) and Ab4 (D) strains. The variance values of PC1 and PC2 are shown in brackets. The ellipses enclose the scores inside a region with 95% confidence. The colors indicate the supernatant samples from untreated (red) and KHS‐Cnd‐treated (green) sessile cultures for each strain and each point represents one of three biological replicates for each supernatant sample. PCA score plots were performed by using the Metaboanalyst 6.0 software.

The PCA score plots showed that the cell‐free supernatants of all *A. baumannii* strains, treated with KHS‐Cnd peptide during biofilm formation, were significantly and markedly distant from the corresponding untreated strains (controls) along the PC1, which represents 87.3%, 83.7%, 88.7%, and 82.7% of the sample variance for ATCC 19606, Ab1, Ab2, and Ab4, respectively (Fig. 1A–D). On the other hand, the supernatant samples of peptide‐treated strains were not detached from their respective controls along the PC2, which accounts for 5.7%, 8.6%, 5.1%, and 7.6% of the sample variance in ATCC 19606, Ab1, Ab2, and Ab4, respectively (Fig. 1A–D).

**Fig. 2:**
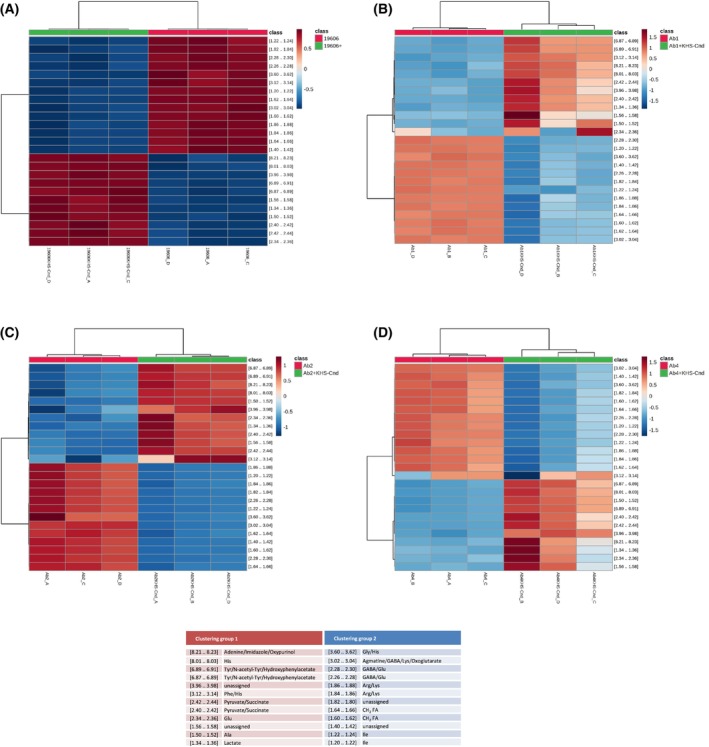
Hierarchical cluster analysis applied on the ^1^H NMR data matrix of *A. baumannii* supernatants after KHS‐Cnd treatment during biofilm formation. Dendrograms (top of the panel) combined with heatmaps of the top 25 ANOVA variables (^1^H NMR buckets) comparing the cell‐free supernatants from KHS‐Cnd treated and untreated sessile cultures of ATCC 19606 (A), Ab1 (B), Ab2 (C) and Ab4 (D) strains.

Furthermore, a hierarchical cluster analysis was performed, and the results are shown in Fig. 2 as Heatmaps, where the top 25 ANOVA variables (^1^H NMR features, namely 0.02 ppm wide window region of the NMR spectra) correspond to rows, while biological replicates for each strain correspond to columns. The heatmaps of free‐cell supernatants from all *A. baumannii* strains showed two distinct clustering groups following the treatment of sessile cultures with KHS‐Cnd (Fig. 2A–D). This analysis confirms that the peptide induces a statistically significant change in the extracellular metabolite profile of biofilm cells for all considered *A. baumannii* strains.

### ^1^H NMR spectra of cell‐free supernatants from sessile cultures of *Acinetobacter baumannii* clinical isolates

After multivariate statistical analysis of the ^1^H NMR data matrix, we attempted to define which extracellular metabolites discriminate the KHS‐Cnd treated samples from control ones, and which are specifically altered in each bacterial supernatant. The analysis of the spectra allowed a partial assignment of the NMR signals. A total of 38 metabolites were identified based on 1D NMR traces (Table S1). Representative ^1^H NMR spectra of supernatant samples from each *A. baumannii* strain are shown in Fig. S3. In Fig. S4, the assigned ^1^H NMR spectral resonances of extracellular metabolites are reported in the following magnified spectral zones: (i) 0.6–2.5 ppm, aliphatic amino acids and organic acids, such as valine and acetate; (ii) 2.5–5.0 ppm, saccharides and other amino or organic acids, such as glucose, methionine, and lactate; (iii) 5.0–9.4 ppm, aromatic amino acids and organic acids, such as tryptophan and formate. From this analysis, we showed that the supernatants from either untreated or KHS‐Cnd‐treated sessile cultures of all considered *A. baumannii* strains contained the same metabolites (Fig. S3).

### Differential analysis of extracellular metabolites of *Acinetobacter baumannii* after KHS‐Cnd treatment during biofilm formation

To compare pairwise the changes observed in extracellular metabolic profiles of the control and the relative KHS‐Cnd‐treated samples, we also performed PLS‐DA (Partial Least Squares Discriminant Analysis). The comparison between treated and untreated *A. baumannii* samples showed a clear separation in two major components that are responsible for: (i) 87.3% and 5.6% of total variance in ATCC 19606; (ii) 83.7% and 7.8% in Ab1; (iii) 88.7% and 4.4% in Ab2; and (iv) 82.6% and 7.0% in Ab4 (Fig. S5).

**Fig. 3:**
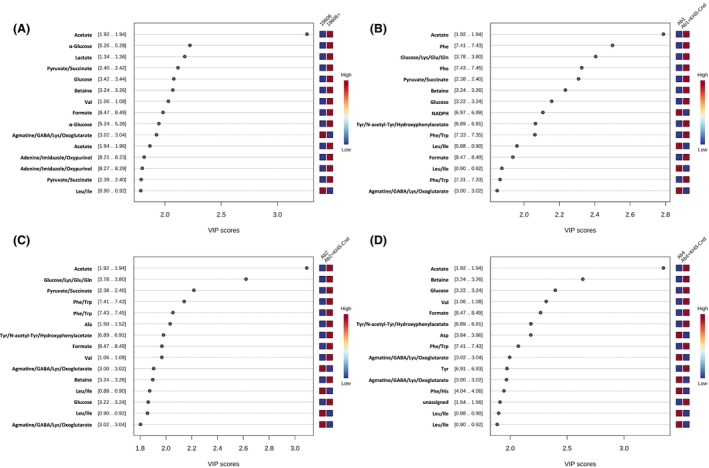
Differential extracellular metabolite analysis of *A. baumannii* clinical isolates treated with KHS‐Cnd peptide during biofilm formation. VIP values of the 15 most important extracellular metabolites in KHS‐Cnd treated and untreated sessile cultures of ATCC 19606 (A), Ab1 (B), Ab2 (C) and Ab4 (D) strains (*P* < 0.05 by Student's *t*‐test).

**Fig. 4:**
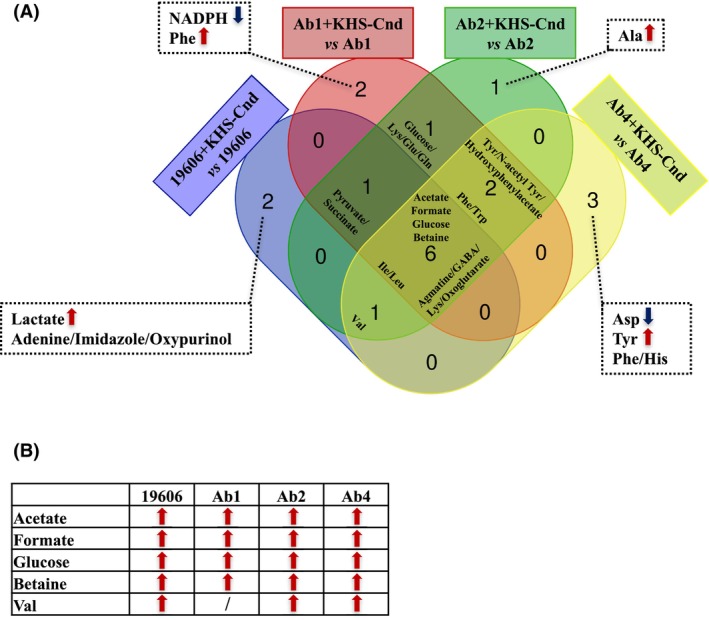
Venn diagram of differentially represented extracellular metabolite analysis of *A. baumannii* clinical isolates treated with KHS‐Cnd peptide during biofilm formation. (A) Venn diagram shows the over‐represented (red arrow) and under‐represented (blue arrow) metabolites specific for each of the four pairwise comparisons (listed in the box) and those common to at least two pairwise comparisons. (B) Within each pairwise comparison, single metabolites shared by *A. baumannii* strains were reported together with their differential representation (KHS‐Cnd treated sessile cultures *versus* untreated ones); / = metabolite was not significantly represented.

After NMR signal assignment, univariate statistical analysis was performed to determine the VIP (Variable Importance in Projection) values representing the most important extracellular metabolites that were differentially represented in KHS‐Cnd treated samples *versus* control ones for each *A. baumannii* strain (Table S2). Fig. 3 reports the VIP values derived from the PLS‐DA analyses and related to the 15 most important metabolites for each pairwise comparison group (Fig. 3A–D). Several extracellular metabolites were significantly more abundant in treated samples than in untreated ones (Fig. 3). In particular, we detected a significant increase of acetate, formate, glucose and betaine in the supernatants of all *A. baumannii* strains upon their exposure to the KHS‐Cnd peptide (Fig. 4A,B). Moreover, the extracellular level of Val was significantly higher for all *A. baumannii* strains except for Ab1 one (Fig. 4A,B).

The VIP variables corresponding to the NMR buckets [0.88..0.90]/[0.90..0.92] and [3.00..3.02]/[3.02..3.04], assigned to the branch‐chain amino acids Ile/Leu and to the group of extracellular metabolites agmatine/GABA/Lys/oxoglutarate, respectively, are significantly under‐represented in all peptide‐treated samples (Fig. 4A). These NMR buckets resulting from more than one metabolite make it challenging to determine the contribution of each individual metabolite and which metabolite is responsible for the statistically significant differences. Moreover, the two groups of extracellular metabolites Phe/Trp and Tyr/N‐acetyl‐Tyr/hydroxyphenylacetate were significantly abundant in the supernatants from all clinical isolates except for the reference strain; the group of metabolites pyruvate/succinate, increased significantly in the supernatants of all strains except for the Ab4 clinical isolate, while the Lys/Glu/Gln group was more represented in the supernatants of Ab1 and Ab2 clinical isolates (Fig. 4A).

On the other hand, we also detected a significant differential representation of strain‐specific extracellular metabolites upon the bacteria's exposure to KHS‐Cnd during biofilm formation. In particular, lactate was more present at extracellular levels only in *A. baumannii* ATCC 19606 reference strain after peptide treatment (Fig. 4A). The metabolites NADPH and Phe were significantly under‐ and over‐represented in the peptide‐treated samples, respectively, only in Ab1 clinical isolate. The extracellular level of Ala, instead, was significantly increased only in *A. baumannii* Ab2 clinical strain; the amino acids Asp and Tyr were less and more present, respectively, only in the supernatants of *A. baumannii* Ab4 clinical isolate (Fig. 4A).

Overall, these analyses showed that the ^1^H NMR‐based approach can reveal extracellular metabolic changes of bacterial pathogen strains during biofilm formation due to the treatment with an antimicrobial peptide, such as KHS‐Cnd, and, therefore, contribute to elucidating the possible antibiofilm effects at a physiological level.

### Pathway analysis of significantly represented extracellular metabolites of *Acinetobacter baumannii* after KHS‐Cnd treatment during biofilm formation

**Fig. 5:**
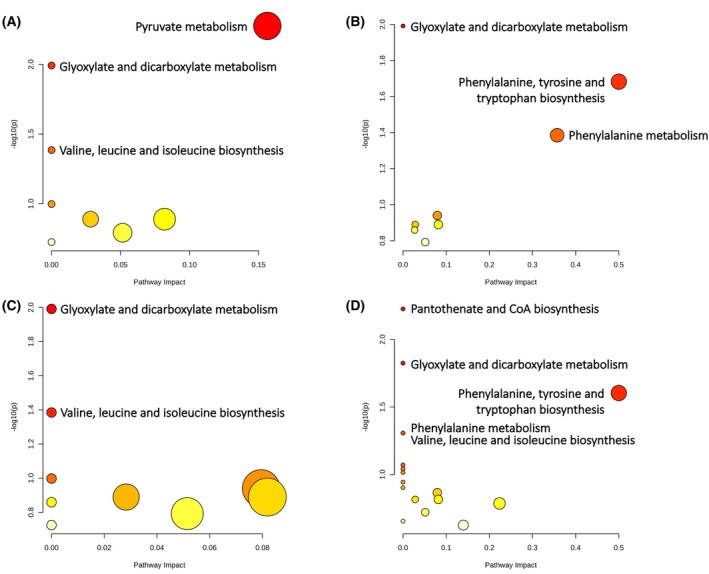
Metabolic pathway enrichment analysis for *A. baumannii* supernatants after KHS‐Cnd treatment during biofilm formation. Metabolic pathways (nodes) are reported according to the *P* and impact values for the cell‐free supernatants from KHS‐Cnd treated and untreated sessile cultures of ATCC 19606 (A), Ab1 (B), Ab2 (C) and Ab4 (D) strains; the size of each node correlates with the number of hits detected within each pathway. For all plots, *P* values range from yellow (less or not significant) to red (more significant, *P* ≤ 0.05) according to Fisher's exact test.

To gain more insights into our obtained biological data, we investigated the strain‐specific differentially represented metabolites by using a pathway enrichment analysis, excluding those metabolite groups assigned to the same NMR signals (Fig. 5). The statistical significance (*P* ≤ 0.05) of the pathway enrichment (KEGG *A. baumannii* database) was plotted against the ‘pathway impact’, which represents the incidence of hit metabolites based on their position within the pathway. A fair number of pathways were found to be significantly involved in KHS‐Cnd‐treated samples *versus* control ones for each studied *A. baumannii* strain (Fig. 5A–D). Metabolites significantly involved in each metabolic pathway are reported in Table S3. On the other hand, the top‐ranking hit ‘glyoxylate and dicarboxylate metabolism’ was shared by all strains within each pairwise comparison (Fig. 5). In all cases, the metabolites significantly enriched within ‘glyoxylate and dicarboxylate metabolism’ were acetate and formate (Table S3), whose extracellular levels were significantly higher in all *A. baumannii* strains (Fig. 4B).

Moreover, ‘pyruvate metabolism’ was the significantly enriched pathway among the differentially represented extracellular metabolites after KHS‐Cnd treatment only for the ATCC 19606 reference strain: interestingly, those found to be significantly enriched within this pathway were acetate and lactate (Table S3), both of which were over‐represented in the supernatants of *A. baumannii* ATCC 19606 strain (Fig. 4). Moreover, ‘Pantothenate and CoA biosynthesis’ was the significantly enriched pathway among the differential extracellular metabolites in KHS‐Cnd treated *versus* untreated sessile cultures only for Ab4 clinical isolate. In the supernatants of this strain, the over‐represented Val and under‐represented Asp were the significantly enriched amino acids belonging to ‘Pantothenate and CoA biosynthesis’ (Table S3 and Fig. 4).

Finally, ‘Valine, leucine and isoleucine biosynthesis’ was shared by the reference strain, Ab2 and Ab4 clinical isolates and Val was the extracellularly over‐represented amino acid to be significantly enriched in this biosynthetic pathway; whereas ‘Phenylalanine, tyrosine and tryptophan biosynthesis’ was shared by Ab1 and Ab4 clinical isolates and, within this pathway, Phe was the significantly enriched amino acid whose extracellular level was increased only for these two strains (Table S3 and Fig. 4).

## Discussion

To date, the clinical management of multidrug‐resistant *A. baumannii* infections has become very challenging. The AMR profile of *A. baumannii* relies on its ability to produce biofilm, whose formation, especially on biotic and abiotic surfaces, is responsible for the mortality and morbidity associated with nosocomial infections [37]. The mechanisms of AMR in biofilms are different from those that confer innate or genetic resistance to bacterial cells under planktonic conditions [38]. Hence, there is a need for new approaches to control and reduce biofilm formation, particularly for those bacteria, such as *A. baumannii*, responsible for chronic infections. Therefore, investigating the impact of antimicrobial drugs on biofilm formation is crucial for the development of effective therapeutic strategies.

In this study, the antibiofilm activity of KHS‐Cnd peptide was investigated by 1D ^1^H NMR analysis on cell‐free supernatants from *A. baumannii* sessile cultures. To this aim, we selected four *A. baumannii* strains, one reference strain (ATCC 19606) and three clinical isolates (Ab1, Ab2, and Ab4). Based on the AMR profile and virulence properties we previously determined, *A. baumannii* ATCC1 19606 is a multidrug‐resistant (MDR) strain, while the other three clinical strains are pandrug resistant (PDR), and all were able to form biofilm [3]. In particular, the KHS‐Cnd peptide was added to the culture medium at sub‐MIC concentrations before adhesion of bacterial cells (pre‐adhesion period, time 0 at 1.5 μm for ATCC 19606, Ab1 and Ab4, and 2.5 μm for Ab2, respectively). This concentration choice is in line with our previously reported results. In fact, the peptide showed MIC values of 5 μm for ATCC 19606, Ab1 and Ab4, and 10 μm for Ab2 and a significant antibiofilm activity on all selected *A. baumannii* strains at concentrations corresponding to 1/4 of MIC values [3]. In particular, we previously demonstrated that KHS‐Cnd impaired biofilm development and caused mature biofilm disaggregation in clinical isolates [3, 13].

To better understand the physiological perturbation induced by KHS‐Cnd peptide on biofilm formation, we investigated the extracellular metabolite profiles through ^1^H NMR analysis. Metabolites related to bacterial anaerobic metabolism, such as acetate and formate, were higher in all tested *A. baumannii* supernatants after KHS‐Cnd treatment. In particular, these fermentation products are common metabolic intermediates in bacterial metabolism that, under anaerobic conditions, can serve as energy substrates for bacterial growth [39]. On the other hand, the secretion of acetate and formate may occur during bacterial growth, thus inhibiting itself after the accumulation of these organic acids in the growth medium [40]. High levels of these fermentation acids, especially in their respective anion forms, can affect bacterial metabolism with an opposite physiological response: formate inhibited most proteins triggered by acetate and several proteins induced by formate are repressed by acetate [41]. Interestingly, these metabolites are able to modulate the expression of proteins involved in biofilm [42, 43]. Moreover, as short‐chain fatty acids, acetate and formate are known to influence interactions between host cells and bacterial pathogens, possibly affecting the host immune responses, thus increasing microbial virulence and contributing to pathogenesis [44, 45]. Notably, our pathway enrichment analysis showed that these two metabolites were significantly enriched within ‘glyoxylate and dicarboxylate metabolism’. This pathway, absent in animals, allows microbial growth on limited carbon sources by producing macromolecules from two‐carbon compounds such as acetate [46, 47]. In addition to this metabolic role, the ‘glyoxylate and dicarboxylate metabolism’ pathway is known to be up‐regulated in *P. aeruginosa* under conditions of antibiotic‐induced oxidative stress and during host infection [48, 49]. Its upregulation in some clinical isolates from urinary tract infections and lungs of cystic fibrosis patients suggests that the glyoxylate shunt is required for pathogenesis and virulence factor production in *P. aeruginosa* and other pathogens [50, 51, 52]. By analyzing the *P. aeruginosa* transcriptome from wound colonization to biofilm wound infection, D'Arpa and colleagues found that ‘glyoxylate and dicarboxylate metabolism’ pathway is upregulated in biofilm infection due to the upregulation of fatty acid degradation whose carbon sources feed the glyoxylate shunt, thus providing the precursors needed to establish infection under oxygen‐ and nutrient‐limited conditions [53]. Moreover, Qi and coworkers demonstrated that *P. aeruginosa* in the biofilm state is more likely to enter a viable but nonculturable state characterized by low metabolic activity where the glyoxylate shunt is required to maintain metabolic homeostasis and bacterial viability [54]. This is consistent with the concept that a reduced central metabolic pathway underlies biofilm metabolism. Therefore, the glyoxylate shunt may be an interesting, yet unexploited drug target, because its inhibition could represent an attractive strategy to control the production of bacterial virulence factors such as biofilm formation. In this study, however, we showed that ‘glyoxylate and dicarboxylate metabolism’ was enriched in the supernatants of biofilm cells upon exposure to the antimicrobial peptide KHS‐Cnd. We hypothesize that defining the extracellular metabolome of KHS‐Cnd‐treated sessile cultures compared to that of untreated *A. baumannii* strains could elucidate the metabolic pathways, such as the glyoxylate shunt, indicative of the impaired biofilm inception/formation.

In addition to the glyoxylate shunt, our NMR analysis showed that the amino acid Val was more present at extracellular levels in three out of four considered *A. baumannii* strains treated with KHS‐Cnd: specifically, ‘valine, leucine and isoleucine biosynthesis’ was the enriched pathway in the supernatants of all treated biofilm cells except in those of the Ab1 strain. Val, a branched‐chain amino acid, plays a controversial role in bacterial growth and biofilm formation, as it can exert both inhibitory and promoting effects on them, depending on the bacteria and specific conditions [55, 56]. Interestingly, some bacterial biofilm cells secrete high levels of Val as a consequence of the metabolic switch occurring during biofilm formation, particularly in carbon‐limited conditions [35, 57].

Overall, high levels of metabolites such as organic acids and amino acids have been reported in *P. aeruginosa* and/or *A. baumannii* biofilms [35, 58]. The typical fermentation products, such as acetate and lactate, may be essential for biofilm formation and long‐term survival in the microaerobic environment of the mature biofilm. Several studies reported that different bacterial species undergo a significant remodeling of energy metabolism during biofilm formation and maintenance [35, 53, 54, 58]: Specifically, glyoxylate and dicarboxylate metabolism was found to satisfy the physiological carbon demand required by the biofilm architecture dictated by a reductive stress condition. This finding is also consistent with the observation that biofilm cells tend to accumulate high levels of amino acids to be used as precursors for energy production. In this regard, Yeom and colleagues reported high levels of Val, Glu, and Lys in biofilm cells of *A. baumannii*1656‐2 strain, supporting the idea that these amino acids accumulate intracellularly to serve as energy substrates necessary for the formation and maintenance of biofilm‐matrix as well as for bacterial survival in the mature biofilm [35, 59].

It is noteworthy that metabolites normally detected inside biofilm cells were found extracellularly after KHS‐Cnd treatment of the considered *A. baumannii* strains. This could be consistent with the antibiofilm activity of KHS‐Cnd peptide that we previously reported in the same *A. baumannii* clinical isolates [3] as well as with the antimembranolytic and antivirulence activities reported in two bacterial models and *P. aeruginosa* clinical isolates, respectively [13, 31]. Further research efforts are needed to understand the mechanisms underlying the peptide's antibiofilm action. First, given that KHS‐Cnd as an AMP is known to induce bacterial membrane disruption, it is plausible that KHS‐Cnd, acting as an antibiofilm peptide, is able to damage biofilm architecture at the level of the extracellular polymeric matrix according to the possible mechanisms of AMPs' action elsewhere described [11, 31]. Then, since KHS‐Cnd is proven to impair biofilm formation and eradicate the already formed biofilms of *A. baumannii* clinical isolates [3], we cannot rule out that this peptide may initially prevent bacterial adhesion and also promote the degradation of protective matrix components in mature biofilms. This can likely occur through KHS‐Cnd‐mediated interference with the QS system and/or downregulating the expression of genes involved in biofilm formation and maintenance. Lastly, all or part of this peptide‐induced scenario may inevitably lead to increased diffusion and low cell density of the biofilms with consequent metabolic remodeling that determines the extracellular accumulation of those key metabolites herein identified as crucial in biofilm formation.

Hence, our ^1^H NMR‐based approach showed that (i) metabolomics analysis at the extracellular level is relatively easier than that at the intracellular level; (ii) differences in extracellular metabolite levels can reflect changes in bacterial metabolism due to peptide treatment; and (iii) determination of extracellular metabolites can also reveal possible different metabolic responses to treatment among the analyzed *A. baumannii* strains.

This study demonstrates that ^1^H NMR analysis of extracellular metabolites during biofilm formation in the presence of an antimicrobial/antivirulence agent is able to highlight the metabolic changes underlying the antibiofilm activity of the drug. Although the lack of information related to the intracellular changes in bacterial cells could be considered as a limitation, in the case of an antimicrobial peptide action specifically related to biofilm formation, the study of the extracellular environment could probably better reflect the response of the bacterial cells as a community. Extracellular metabolic analysis could be considered as a signature of biofilm state. Therefore, the identification of biofilm‐associated extracellular metabolites, which could serve as biomarkers of the bacterial biofilm state, could be of pivotal importance as molecular targets of new antibiotics or drugs and, finally, they could be also used as diagnostic tools to assay their potential therapeutic efficacy. In conclusion, this study dissected the biological implications of the observed changes in metabolic pathways upon the exposure of biofilm cells to an AMP, thus contributing to lay the foundations for the development of new treatments against biofilm‐based infections that are difficult to eradicate. Given the importance of exploring the specific physiological traits of bacterial biofilms, new antimicrobial targets can be developed by identifying the metabolic pathways, such as glyoxylate and dicarboxylate metabolism and branched‐chain amino acid biosynthesis, which, when altered in any way, will inhibit biofilm formation and maintenance. Furthermore, it cannot be ruled out that, once these antimicrobial targets have been identified, the synergistic effects of the AMP of interest with conventional antimicrobial agents could also be evaluated to optimize their therapeutic efficacy.

## Conflict of interest

The authors declare no conflict of interest.

## Author contributions

FP designed and performed NMR analysis and wrote the manuscript. EL performed multivariate statistical analysis. FM prepared samples for NMR analysis. IP performed the bacterial cultures and peptide treatment. RP wrote the manuscript and supervised the experiments. MA reviewed and revised the manuscript. LS reviewed and revised the manuscript. SB performed and validated multivariate statistical analysis. FB acquired funding and wrote the manuscript. EI conceived and supervised the experiments and wrote the manuscript. All authors read and approved the final manuscript.

## Supporting information


**Fig. S1.** Certificate of analysis of KHS‐Cnd peptide.
**Fig. S2.** 400 MHz ^1^H/^1^H 2D TOCSY NMR spectra of cell‐free supernatants from *A. baumannii* sessile cultures.
**Fig. S3.** 400 Mhz 1D ^1^H NMR spectral traces of all cell‐free supernatants from *A. baumannii* sessile cultures.
**Fig. S4.** 400 Mhz 1D ^1^H NMR spectral representative traces of cell‐free supernatants from *A. baumannii* sessile cultures.
**Fig. S5.** Multivariate statistical analysis applied on the ^1^H NMR data matrix of *A. baumannii* supernatants after KHS‐Cnd treatment during biofilm formation.
**Table S1.** ^1^H NMR signal assignments.
**Table S2.** ^1^H NMR variables with VIP score > 1 and *P* < 0.05 corresponding to extracellular metabolites sensitive to KHS‐Cnd peptide treatment in ATCC 19606, Ab1, Ab2 and Ab4 strains.
**Table S3.** Results of pathway analysis of ^1^H NMR metabolomics data.

## Data Availability

The data that support the findings of this study are available from the corresponding authors (fbuono@unitus.it; imperlini@unitus.it) upon reasonable request.
