# Supplementary material for: Antibiofilm activity of a chionodracine‐derived peptide by NMR‐based metabolomics of cell‐free supernatant of Acinetobacter baumannii clinical strains
Source: FEBS Open Bio. 2025 Nov 2;16(4):751–65. doi: 10.1002/2211-5463.70156 (PMC13042655; doi:10.1002/2211-5463.70156)
Supplement: Supplementary file 1 — Fig. S1. Certificate of analysis of KHS‐Cnd peptide. Fig. S2. 400 MHz 1H/1H 2D TOCSY NMR spectra of cell‐free supernatants from A. baumannii sessile cultures. Fig. S3. 400 Mhz 1D 1H NMR spectral traces of all cell‐free supernatants from A. baumannii sessile cultures. Fig. S4. 400 Mhz 1D 1H NMR spectral representative traces of cell‐free supernatants from A. baumannii sessile cultures. Fig. S5. Multivariate statistical analysis applied on the 1H NMR data matrix of A. baumannii supernatants after KHS‐Cnd treatment during biofilm formation. Table S1. 1H NMR signal assignments. Table S2. 1H NMR variables with VIP score > 1 and P < 0.05 corresponding to extracellular metabolites sensitive to KHS‐Cnd peptide treatment in ATCC 19606, Ab1, Ab2 and Ab4 strains. Table S3. Results of pathway analysis of 1H NMR metabolomics data. [file FEB4-16-751-s001.pdf]

Figure S1

Certificate of Analysis

For research use only!

Product:	Synthetic peptide
Lot No.:	P220118-03-01
Manufacturing date:	September 28th 2018
Peptide Sequence:	WFGKLYRGITKVVKKVKGLLKG
Modifications:	None.
Format:	Lyophilized trifluoroacetate salt.
Theoretical M.W. of peptide:	2519.19
M.W. (M.W. + H^+) Measured by MS:	2520.42
M.W. Measured by MS:	2519.42
Match:	Approved.
Purity:	99.76%
Delivered quantity:	25.0 mg
HPLC results:	Enclosed.
Mass spec. results:	Enclosed.

CASLO ApS, Diplomvej 381, DK-2800 Lyngby, Denmark
Tel: +45- 70 23 28 60, Fax: +45- 70 23 29 90
e-mail: info@caslo.com Web page: www.caslo.com

Figure S1-continued

CASLO

Storage and shelf life

Store the peptide at -20^{0}C. It is recommended to store the peptide in lyophilized format. If the peptide is stored for less than 48 hours before use the peptide can be stored at 5^{0}C (or room temperature). Do not repeatedly thaw and freeze. It is recommended to use the peptide before 1 year after the manufacturing date.

Hydrophobicity of sequence composition

Hydrophobic amino acids: 40.91%
Acidic amino acids: 0.00%
Basic amino acids: 31.82%
Neutral amino acids: 27.27%

Figure S1-continued

HPLC Certificate

**Lot No.**	P220118-03-01
**HPLC Column**	(250×4.6mm I.D.) C18
**Detection wavelength**	220 nm
**Gradient**	25-40%B in 15 min
**Buffer A**	0.05%TFA +2%CH_3CN
**Buffer B**	0.05%TFA +90%CH_3CN

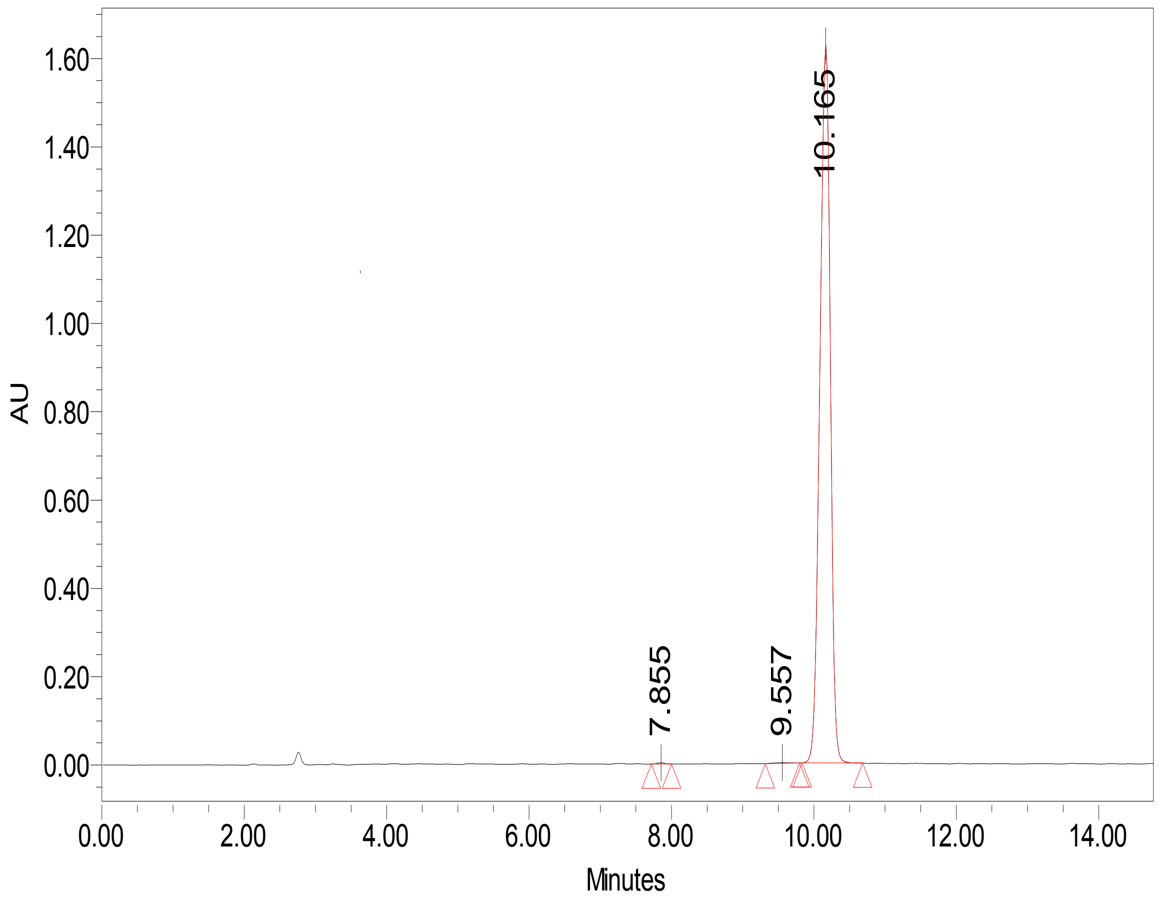

	Retention Time	Area	% Area	Height
1	7.855	19416	0.12	2703
2	9.557	20245	0.12	1398
3	10.165	16545583	99.76	1626777

Figure S1-continued

Mass Spectrometry Certificate

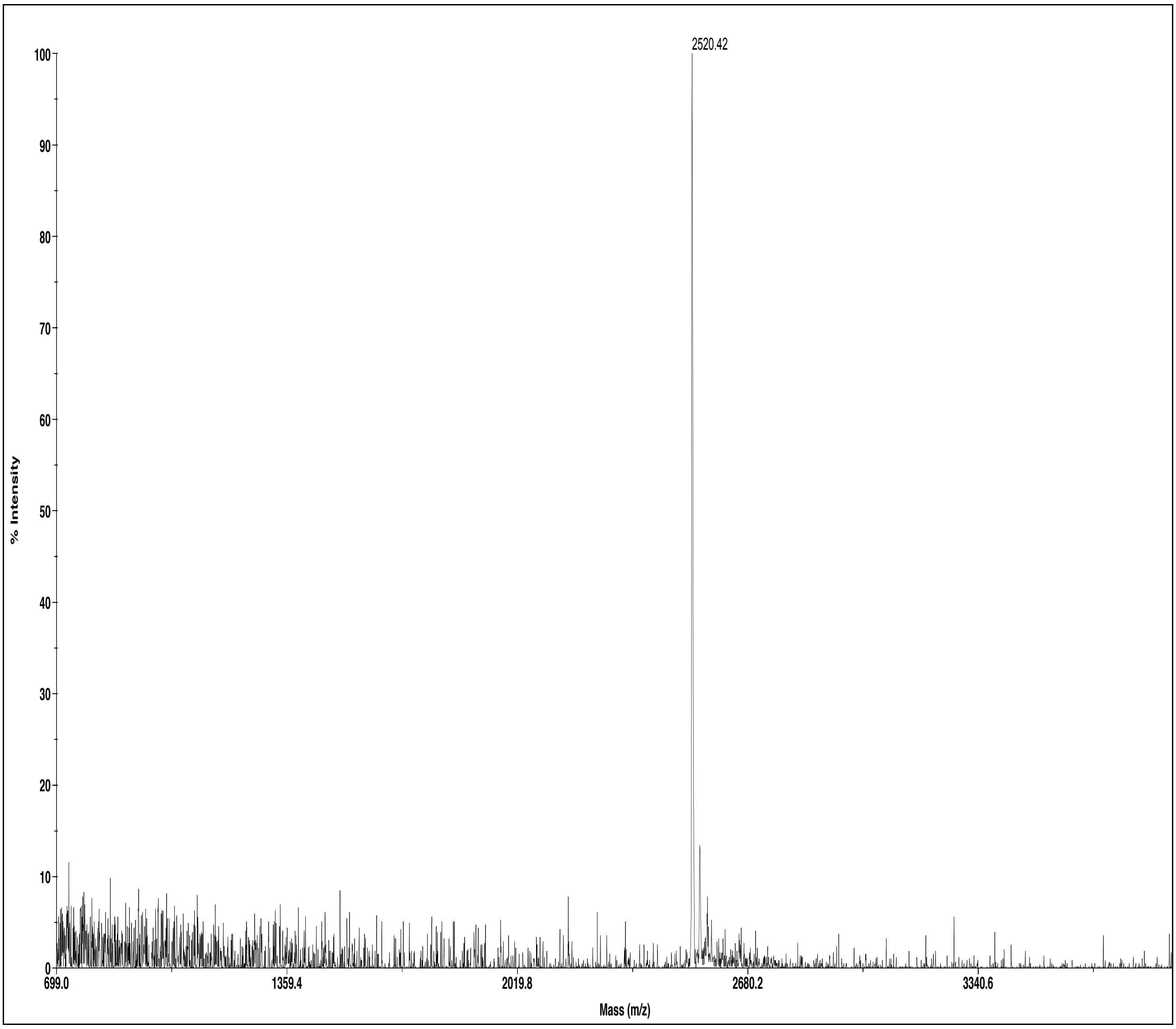

Lot No.: P220118-03-01
Method: MALDI-TOF
Main Peak: 2520.42
MW [M+H$^+$]: 2520.42
MW: 2519.42
Theoretical MW: 2519.19
Match: Approved
Z=1

Figure S2

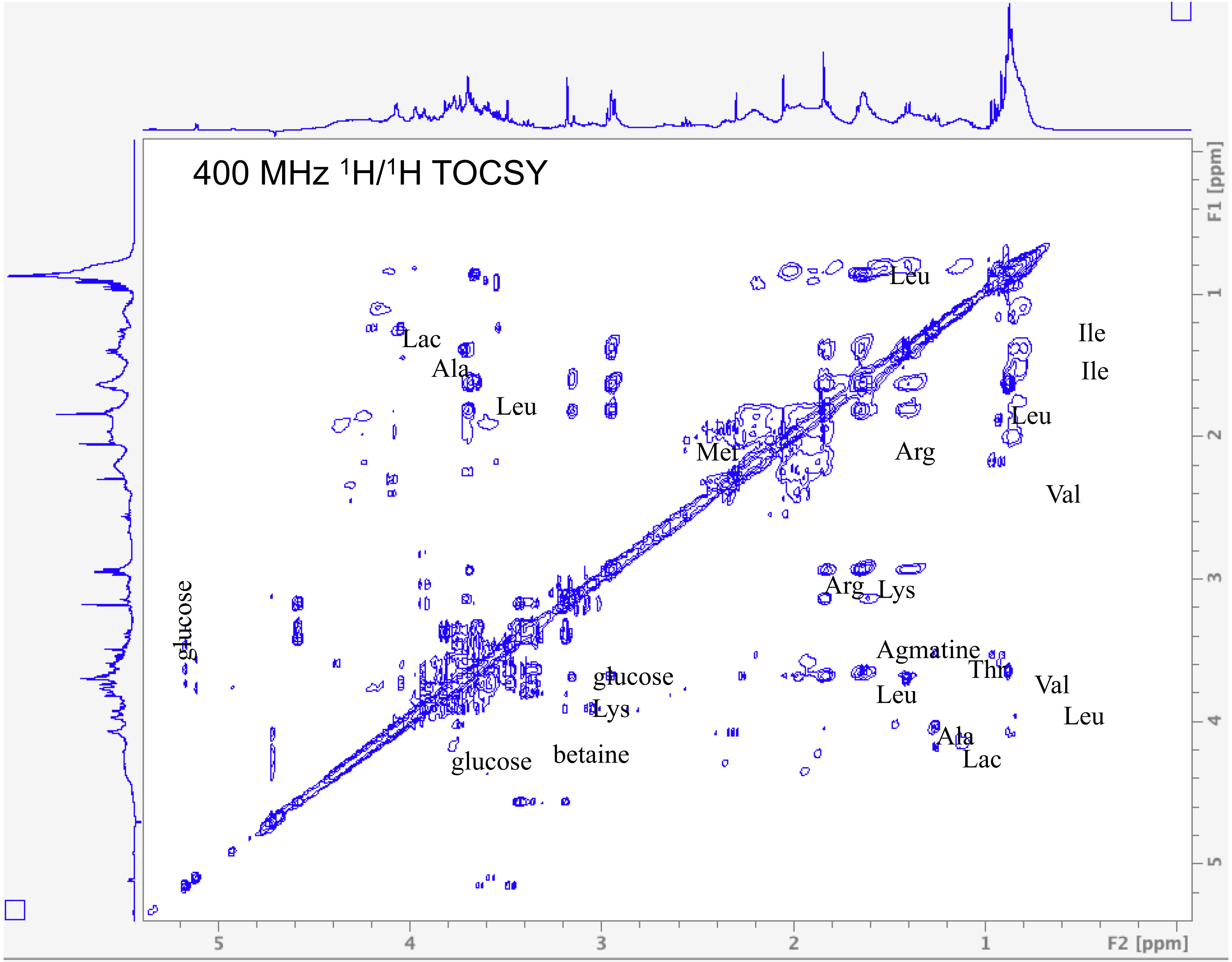

Figure S2. 400 MHz $^1H/^1H$ 2D TOCSY NMR spectra of cell-free supernatants from *A. baumannii* sessile cultures.

Table S1. ^{1}H NMR signal assignments.

KEGG identifier	Metabolite	Chemical shift [ppm]
C00033	**Acetate**	1.93 (s)
C00147	**Adenine**	8.23 (d)
C00179	**Agmatine**	1.70 (m)<br>3.05 (t)
C00041	**Alanine**	1.50 (d)<br>3.75 (q)
C00062	**Arginine**	1.70 (m)<br>1.86 (m)<br>3.05 (m)
C00049	**Aspartate**	3.85 (dd)
C00719	**Betaine**	3.25 (s)<br>3.89 (s)
C00114	**Choline**	3.30 (s)
C00058	**Formate**	8.48 (s)
C00031	**Glucose**	3.23 (m)<br>3.46 (m)<br>5.21 (d)<br>5.25 (d)
C00025	**Glutamate**	2.10 (m)<br>2.36 (m)<br>3.80 (t)
C00064	**Glutamine**	3.80 (t)
C00037	**Glycine**	3.60 (s)
C00135	**Histidine**	3.05 (d)<br>4.05 (t)<br>7.20 (s)<br>8.00 (s)
C05984	**Hydroxybutyrate**	4.30 (m)
C00642	**Hydroxyphenylacetate**	6.86 (d)
C05568	**Imidazole**	8.25 (s)
C00407	**Isoleucine**	0.92 (t)<br>1.00 (d)<br>1.22 (m)<br>3.70 (d)
C00256	**Lactate**	1.35 (d)<br>4.15 (q)
C00123	**Leucine**	0.92 (m)<br>1.76 (m)<br>3.75 (m)
C00047	**Lysine**	1.70 (m)<br>3.05 (t)<br>3.80 (t)
C00073	**Methionine**	2.15 (s)<br>2.65 (t)
C00082	**N-acetyltyrosine**	6.90 (d)
C00003	**NAD^{+}**	5.90 (d)
C00005	**NADPH**	6.40 (d)<br>6.99 (s)

C00026	**Oxoglutarate**	3.05 (t)
C07599	**Oxypurinol**	8.23 (s)
C00079	**Phenylalanine**	3.15 (m)<br>4.05 (d)<br>7.40 (m)
C00022	**Pyruvate**	2.40 (s)
C00042	**Succinate**	2.40 (s)
C00089	**Sucrose**	5.43 (m)
C00188	**Threonine**	3.60 (d)
C00078	**Tryptophan**	7.20 (d)<br>7.35 (m)<br>7.70 (d)
C00082	**Tyrosine**	6.90 (d)<br>7.20 (d)
C00105	**UMP**	5.90 (m)
C00299	**Uridine**	5.90 (d)
C00183	**Valine**	1.05 (d)<br>2.21 (m)<br>3.56 (d)
C00334	**4-Aminobutyrate (GABA)**	2.30 (t)<br>3.05 (t)

Figure S3

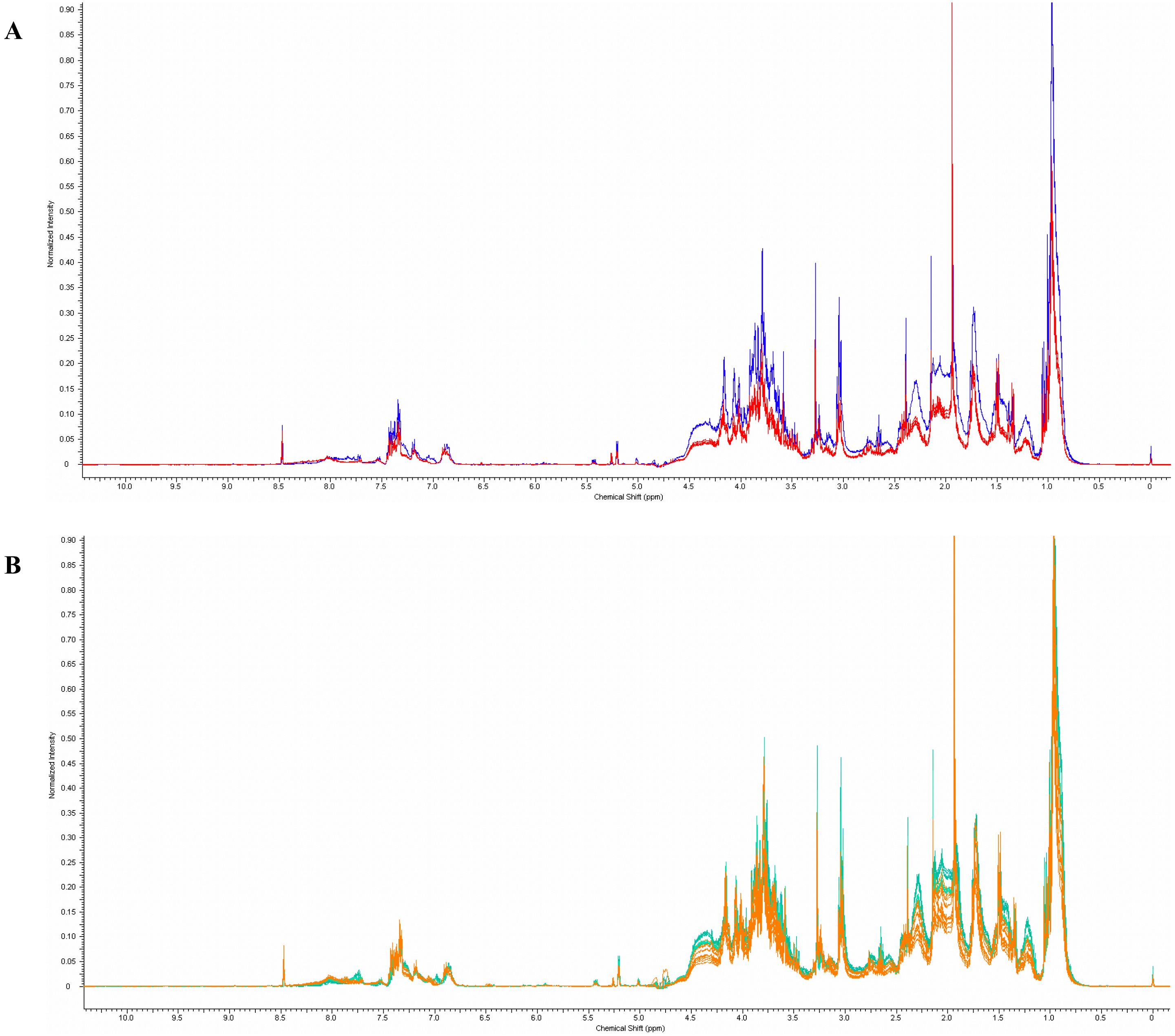

Figure S3. 400 Mhz 1D ^{1}H NMR spectral traces of all cell-free supernatants from *A. baumannii* sessile cultures. ^{1}H NMR spectrum of cell-free supernatants from (A) untreated (blue) and KHS-Cnd treated (red) sessile cultures of ATCC 19606 reference strain and (B) untreated (cyano) and KHS-Cnd treated (orange) sessile cultures of Ab1, Ab2, Ab4 clinical strains.

Figure S4

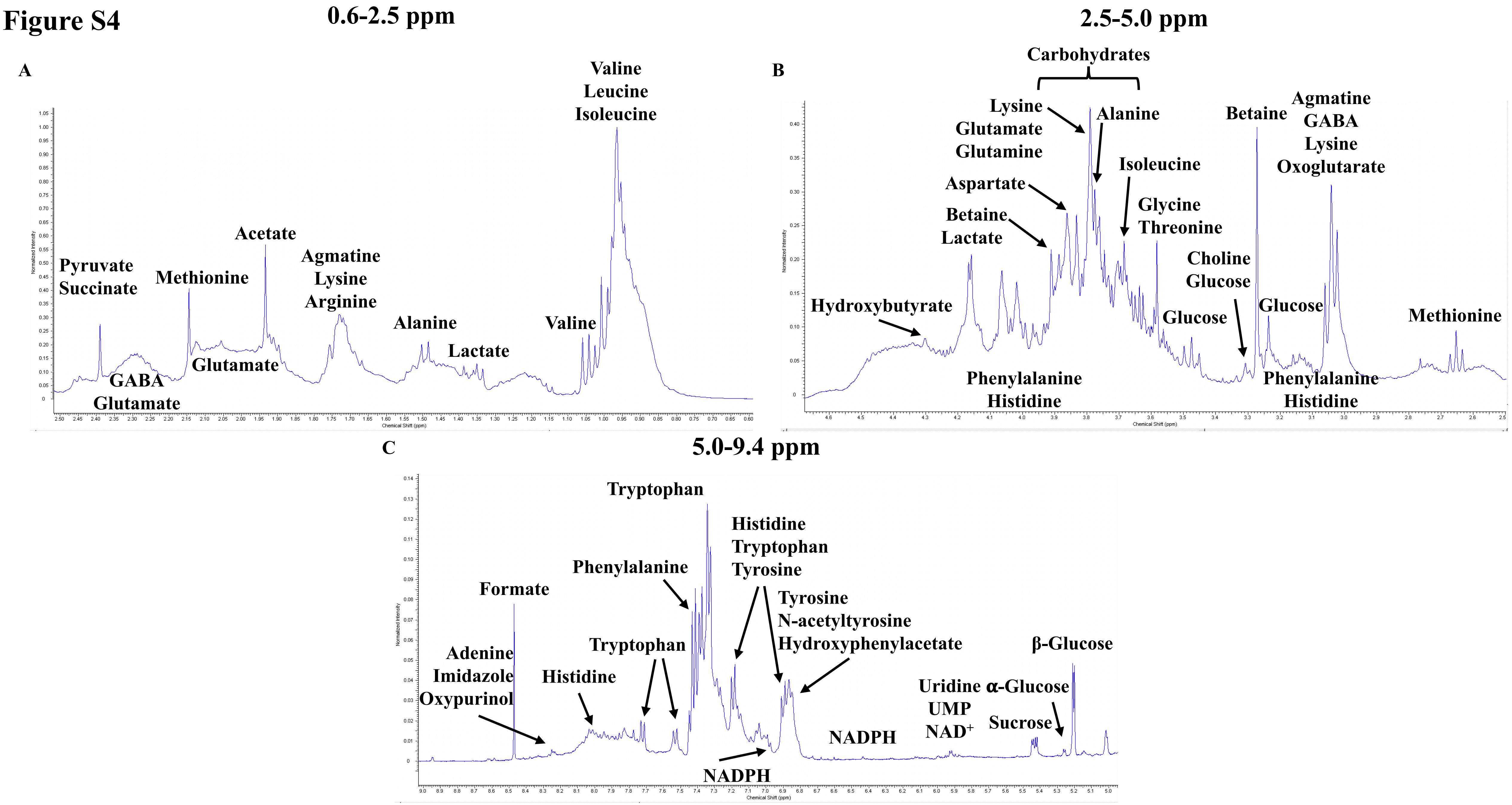

Figure S4. 400 Mhz 1D ^{1}H NMR spectral representative traces of cell-free supernatants from *A. baumannii* sessile cultures. ^{1}H NMR spectrum of cell-free supernatants from untreated sessile cultures of ATCC 19606 is reported in 0.6-2.5 ppm (A) 2.5-5.0 ppm (B) 5.0-9.4 ppm (C) magnified spectral zones.

Figure S5

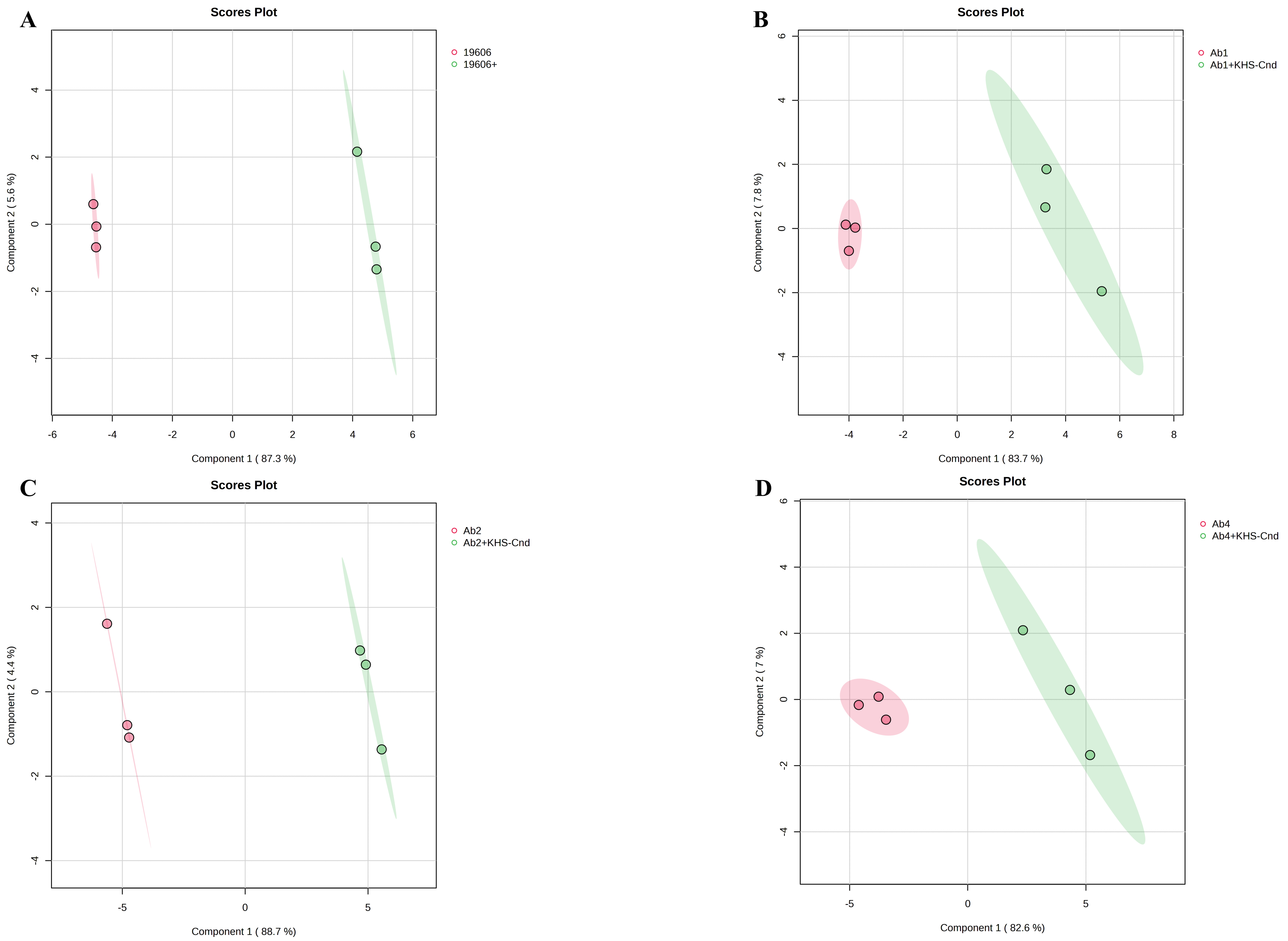

Figure S5. Multivariate statistical analysis applied on the ^{1}H NMR data matrix of *A. baumannii* supernatants after KHS-Cnd treatment during biofilm formation. PLS-DA score-plot of cell-free supernatants from untreated and KHS-Cnd treated sessile cultures of ATCC 19606 (A), Ab1 (B), Ab2 (C) and Ab4 (D) strains. Values of the two major components of total variance are shown in brackets. The ellipses enclose the scores inside a region with 95% confidence. The colors indicate the supernatant samples from untreated (red) and KHS-Cnd treated (green) sessile cultures for each strain and each point represents one of three biological replicates for each supernatant sample. PCA score-plots were performed by using Metaboanalyst 6.0 software.

Table S2. ^{1}H NMR variables with VIP score > 1 and $p < 0.05$ corresponding to extracellular metabolites sensitive to KHS-Cnd peptide treatment in ATCC 19606, Ab1, Ab2 and Ab4 strains.

Chemical shift [ppm]	Metabolite	VIP score	*p* value
	**19606+KHS-Cnd vs 19606**		
[1.92 .. 1.94]	Acetate	3.26	3.38e-06
[5.26 .. 5.28]	α-Glucose	2.22	1.76e-05
[1.34 .. 1.36]	Lactate	2.17	2.09e-07
[2.40 .. 2.42]	Pyruvate/Succinate	2.12	1.37e-06
[3.42 .. 3.44]	Glucose	2.08	1.57e-04
[3.24 .. 3.26]	Betaine	2.07	1.11e-05
[1.06 .. 1.08]	Val	2.03	7.12e-05
[8.47 .. 8.49]	Formate	1.98	3.00e-04
[5.24 .. 5.26]	α-Glucose	1.95	1.57e-04
[3.02 .. 3.04]	Agmatine/GABA/Lys/Oxoglutarate	1.92	1.15e-06
[1.94 .. 1.96]	Acetate	1.87	8.36e-04
[8.21 .. 8.23]	Adenine/Imidazole/Oxypurinol	1.81	1.39e-06
[8.27 .. 8.29]	Adenine/Imidazole/Oxypurinol	1.80	3.35e-06
[2.38 .. 2.40]	Pyruvate/Succinate	1.79	4.51e-05
[0.90 .. 0.92]	Leu/Ile	1.78	6.43e-05
[3.44 .. 3.46]	Glucose	1.74	2.17e-04
[0.88 .. 0.90]	Ile	1.72	4.20e-05
[3.28 .. 3.30]	Choline/Glucose	1.72	5.45e-05
[0.92 .. 0.94]	Leu/Ile	1.70	6.05e-05
[0.94 .. 0.96]	Val/Leu/Ile	1.70	4.94e-05
[7.55 .. 7.57]	Trp	1.69	3.92e-06
[3.68 .. 3.70]	Ile	1.68	1.39e-05
[6.99 .. 7.01]	NADPH	1.64	5.12e-06
[6.89 .. 6.91]	Tyr/N-acetyl-Tyr/Hydroxyphenylacetate	1.60	6.15e-09
[1.50 .. 1.52]	Ala	1.59	4.97e-07
[8.25 .. 8.27]	Adenine/Imidazole/Oxypurinol	1.55	1.02e-05
[0.86 .. 0.88]	Ile	1.55	3.15e-04
[8.03 .. 8.05]	His	1.54	1.94e-06
[4.06 .. 4.08]	Phe/His	1.53	3.43e-03
[3.80 .. 3.82]	Glu/Gln	1.50	3.54e-04
[4.04 .. 4.06]	Phe/His	1.49	8.67e-05
[1.32 .. 1.34]	Lactate	1.48	1.20e-03
[8.23 .. 8.25]	Adenine/Imidazole/Oxypurinol	1.47	1.47e-05
[3.22 .. 3.24]	Glucose	1.45	7.47e-05
[5.18 .. 5.20]	β-Glucose	1.45	2.44e-04
[3.26 .. 3.28]	Betaine	1.44	1.62e-04
[1.70 .. 1.72]	Agmatine/Lys/Arg	1.41	1.80e-05
[1.48 .. 1.50]	Ala	1.40	3.00e-06
[3.78 .. 3.80]	Lys	1.39	6.40e-03

[3.46 .. 3.48]	Glucose	1.38	1.46e-03
[3.48 .. 3.50]	Glucose	1.37	1.54e-03
[4.14 .. 4.16]	Lactate	1.35	5.85e-05
[3.30 .. 3.32]	Choline/Glucose	1.30	5.74e-05
[1.68 .. 1.70]	Agmatine/Lys/Arg	1.29	5.47e-06
[8.01 .. 8.03]	His	1.27	4.50e-07
[1.00 .. 1.02]	Val	1.24	1.20e-04
[2.28 .. 2.30]	GABA/Glu	1.20	8.93e-07
[7.99 .. 8.01]	His	1.18	2.15e-06
[1.66 .. 1.68]	Agmatine/Lys/Arg	1.17	2.74e-06
[6.87 .. 6.89]	Tyr/N-acetyl-Tyr/Hydroxyphenylacetate	1.16	6.71e-07
[2.26 .. 2.28]	GABA/Glu	1.15	8.48e-07
[6.97 .. 6.99]	NADPH	1.14	2.40e-04
[3.58 .. 3.60]	Gly/Thr	1.11	5.23e-04
[2.30 .. 2.32]	GABA/Glu	1.10	1.83e-06
[2.62 .. 2.64]	Met	1.05	4.51e-05
[7.41 .. 7.43]	Phe	1.04	1.72e-05
[7.19 .. 7.21]	His/Trp/Tyr	1.03	1.60e-04
[2.64 .. 2.66]	Met	1.01	4.38e-05
**Ab1+KHS-Cnd *vs* Ab1**			
[1.92 .. 1.94]	Acetate	2.79	5.24e-03
[7.41 .. 7.43]	Phe	2.50	1.69e-05
[3.78 .. 3.80]	Glucose/Lys/Glu/Gln	2.41	2.08e-03
[7.43 .. 7.45]	Phe	2.33	3.21e-05
[2.38 .. 2.40]	Pyruvate/Succunate	2.31	1.39e-03
[3.24 .. 3.26]	Betaine	2.23	6.49e-05
[3.22 .. 3.24]	Glucose	2.15	4.60e-05
[6.97 .. 6.99]	NADPH	2.11	1.15e-04
[6.89 .. 6.91]	Tyr/N-acetyl-Tyr/Hydroxyphenylacetate	2.06	4.48e-04
[7.33 .. 7.35]	Phe/Trp	2.06	5.87e-05
[0.88 .. 0.90]	Leu/Ile	1.96	1.52e-03
[8.47 .. 8.49]	Formate	1.94	4.80e-03
[0.90 .. 0.92]	Leu/Ile	1.87	2.18e-03
[7.31 .. 7.33]	Phe/Trp	1.86	1.82e-05
[3.00 .. 3.02]	Agmatine/GABA/Lys/Oxoglutarate	1.85	3.34e-03
[3.02 .. 3.04]	Agmatine/GABA/Lys/Oxoglutarate	1.81	1.62e-03
[7.75 .. 7.77]	Trp	1.77	6.58e-07
[0.86 .. 0.88]	Ile	1.68	9.32e-03
[0.92 .. 0.94]	Leu/Ile	1.67	7.86e-03
[7.55 .. 7.57]	Trp	1.65	1.15e-03
[4.06 .. 4.08]	Phe/His	1.63	5.88e-03
[8.01 .. 8.03]	His	1.59	1.03e-03
[7.39 .. 7.41]	Phe	1.58	7.60e-06
[7.99 .. 8.01]	His	1.58	3.46e-05

[2.28 .. 2.30]	GABA/Glu	1.58	5.75e-05
[3.58 .. 3.60]	Gly/Thr	1.58	6.73e-04
[2.40 .. 2.42]	Pyruvate/Succinate	1.58	3.81e-03
[3.30 .. 3.32]	Choline/Glucose	1.58	4.95e-04
[8.03 .. 8.05]	His	1.56	1.44e-03
[1.50 .. 1.52]	Ala	1.54	1.17e-02
[3.04 .. 3.06]	Agmatine/GABA/Lys/Oxoglutarate	1.52	7.14e-03
[8.21 .. 8.23]	Adenine/Imidazole/Oxypurinol	1.49	1.53e-03
[8.27 .. 8.29]	Adenine/Imidazole/Oxypurinol	1.48	1.67e-03
[4.16 .. 4.18]	Lactate	1.45	1.87e-02
[2.26 .. 2.28]	GABA/Glu	1.42	3.61e-04
[7.19 .. 7.21]	His/Trp/Tyr	1.41	1.36e-03
[3.28 .. 3.30]	Choline/Glucose	1.40	4.20e-04
[2.30 .. 2.32]	GABA/Glu	1.35	1.61e-04
[8.23 .. 8.25]	Adenine/Imidazole/Oxypurinol	1.32	8.78e-04
[1.02 .. 1.04]	Val	1.31	2.89e-03
[6.87 .. 6.89]	Tyr/N-acetyl-Tyr/Hydroxyphenylacetate	1.31	8.77e-04
[1.48 .. 1.50]	Ala	1.28	4.06e-04
[1.00 .. 1.02]	Val	1.28	1.16e-03
[7.37 .. 7.39]	Phe	1.22	2.39e-05
[4.00 .. 4.02]	Phe/His	1.21	2.42e-03
[3.14 .. 3.16]	Phe	1.20	4.68e-06
[1.32 .. 1.34]	Lactate	1.20	7.35e-03
[3.60 .. 3.62]	Gly/His	1.18	2.13e-04
[1.34 .. 1.36]	Lactate	1.13	3.26e-03
[3.70 .. 3.72]	Ile	1.13	5.34e-03
[2.64 .. 2.66]	Met	1.10	6.35e-04
[2.10 .. 2.12]	Glu	1.06	1.77e-03
[7.69 .. 7.71]	Trp	1.05	1.30e-03
[4.30 .. 4.32]	Hydroxybutyrate	1.02	5.99e-04
[2.08 .. 2.10]	Glu	1.01	3.02e-04
**Ab2+KHS-Cnd *vs* Ab2**			
[1.92 .. 1.94]	Acetate	3.09	2.79e-05
[3.78 .. 3.80]	Glucose/Lys/Glu/>gln	2.62	2.39e-04
[2.38 .. 2.40]	Pyruvate/Succinate	2.21	1.88e-04
[7.41 .. 7.43]	Phe/Trp	2.14	4.22e-04
[7.43 .. 7.45]	Phe/Trp	2.05	6.79e-04
[1.50 .. 1.52]	Ala	2.03	1.90e-06
[6.89 .. 6.91]	Tyr/N-acetyl-Tyr/Hydroxyphenylacetate	1.98	4.09e-05
[8.47 .. 8.49]	Formate	1.97	3.76e-06
[1.06 .. 1.08]	Val	1.97	1.75e-06
[3.00 .. 3.02]	Agmatine/GABA/Lys/Oxoglutarate	1.90	1.99e-06
[3.24 .. 3.26]	Betaine	1.90	1.06e-05
[0.88 .. 0.90]	Leu/Ile	1.87	6.84e-06

[3.22 .. 3.24]	Glucose	1.86	5.42e-06
[0.90 .. 0.92]	Leu/Ile	1.86	7.99e-06
[3.02 .. 3.04]	Agmatine/GABA/Lys/Oxoglutarate	1.80	2.30e-06
[0.92 .. 0.94]	Leu/Ile	1.78	9.64e-06
[4.06 .. 4.08]	Phe/His	1.78	8.43e-05
[7.33 .. 7.35]	Trp	1.77	7.28e-05
[6.97 .. 6.99]	NADPH	1.76	2.92e-07
[2.40 .. 2.42]	Pyruvate/Succinate	1.73	1.07e-04
[0.86 .. 0.88]	Ile	1.72	2.60e-05
[4.16 .. 4.18]	Lactate	1.70	3.42e-05
[7.55 .. 7.57]	Trp	1.69	5.76e-04
[3.80 .. 3.82]	Glu/Gln	1.68	2.81e-06
[8.03 .. 8.05]	His	1.64	9.87e-04
[3.74 .. 3.76]	Ala	1.60	3.27e-04
[8.21 .. 8.23]	Adenine/Imidazole/Oxypurinol	1.60	2.62e-04
[8.01 .. 8.03]	His	1.60	3.23e-04
[8.27 .. 8.29]	Adenine/Imidazole/Oxypurinol	1.58	1.66e-03
[7.99 .. 8.01]	His	1.55	3.39e-04
[5.18 .. 5.20]	beta-Glucose	1.51	3.19e-06
[2.28 .. 2.30]	GABA/Glu	1.51	1.22e-05
[2.26 .. 2.28]	GABA/Glu	1.49	6.57e-05
[0.94 .. 0.96]	Val/Leu/Ile	1.47	4.29e-05
[3.58 .. 3.60]	Gly/Thr	1.47	5.34e-05
[1.48 .. 1.50]	Ala	1.46	1.23e-03
[7.75 .. 7.77]	Trp	1.44	1.05e-06
[1.34 .. 1.36]	Lactate	1.44	2.72e-04
[3.30 .. 3.32]	Choline/Glucose	1.40	6.61e-06
[6.99 .. 7.01]	NADPH	1.39	8.31e-05
[7.39 .. 7.41]	Phe	1.38	1.70e-03
[6.87 .. 6.89]	Tyr/N-acetyl-Tyr/Hydroxyphenylacetate	1.36	1.51e-04
[8.23 .. 8.25]	Adenine/Imidazole/Oxypurinol	1.35	6.71e-04
[4.00 .. 4.02]	Phe/His	1.31	3.31e-04
[7.19 .. 7.21]	His/Trp/Tyr	1.30	7.49e-06
[3.60 .. 3.62]	Gly/His	1.30	9.55e-04
[2.30 .. 2.32]	GABA/Glu	1.24	2.62e-05
[3.28 .. 3.30]	Choline/Glucose	1.23	2.19e-04
[3.04 .. 3.06]	Agmatine/GABA/Lys/Oxoglutarate	1.18	2.72e-04
[1.00 .. 1.02]	Val	1.16	3.98e-04
[1.94 .. 1.96]	Acetate	1.15	2.81e-06
[2.64 .. 2.66]	Met	1.11	2.40e-04
[8.25 .. 8.27]	Adenine/Imidazole/Oxypurinol	1.10	5.19e-04
[2.10 .. 2.12]	Glu	1.04	3.44e-05
[4.30 .. 4.32]	Hydroxybutyrate	1.04	1.95e-05
[2.62 .. 2.64]	Met	1.04	2.86e-04

[1.66 .. 1.68]	Agmatine/Lys/Arg	1.03	2.65e-05
[7.37 .. 7.39]	Phe	1.03	2.76e-03
[1.02 .. 1.04]	Val	1.02	9.97e-05
**Ab4+KHS-Cnd *vs* Ab4**			
[1.92 .. 1.94]	Acetate	3.34	1.99e-03
[3.24 .. 3.26]	Betaine	2.63	7.81e-05
[3.22 .. 3.24]	Glucose	2.39	7.69e-05
[1.06 .. 1.08]	Val	2.31	2.54e-04
[8.47 .. 8.49]	Formate	2.26	4.43e-04
[6.89 .. 6.91]	Tyr/N-acetyl-Tyr/Hydroxyphenylacetate	2.18	4.93e-04
[7.41 .. 7.43]	Phe/Trp	2.07	2.72e-04
[3.02 .. 3.04]	Agmatine/GABA/Lys/Oxoglutarate	1.99	5.33e-04
[3.00 .. 3.02]	Agmatine/GABA/Lys/Oxoglutarate	1.96	4.77e-04
[4.04 .. 4.06]	Phe/His	1.94	3.64e-05
[1.54 .. 1.56]	unassigned	1.91	6.21e-05
[0.88 .. 0.90]	Leu/Ile	1.90	6.78e-04
[0.90 .. 0.92]	Leu/Ile	1.88	8.14e-04
[2.40 .. 2.42]	Pyruvate/Succinate	1.87	1.00e-02
[3.80 .. 3.82]	Glu/Gln	1.81	8.21e-04
[4.14 .. 4.16]	Lactate	1.79	6.07e-05
[0.86 .. 0.88]	Ile	1.79	5.94e-04
[7.33 .. 7.35]	Trp	1.78	2.30e-04
[1.50 .. 1.52]	Ala	1.78	5.46e-04
[0.92 .. 0.94]	Leu/Ile	1.72	2.21e-03
[1.48 .. 1.50]	Ala	1.71	3.11e-06
[5.18 .. 5.20]	beta-Glucose	1.70	1.64e-04
[0.94 .. 0.96]	Val/Leu/Ile	1.66	1.92e-03
[6.97 .. 6.99]	NADPH	1.65	4.14e-05
[7.19 .. 7.21]	His/Trp/Tyr	1.61	1.94e-04
[6.99 .. 7.01]	NADPH	1.59	1.80e-02
[1.94 .. 1.96]	Acetate	1.59	5.29e-03
[4.16 .. 4.18]	Lactate	1.50	4.11e-04
[3.74 .. 3.76]	Ala	1.44	5.24e-04
[3.28 .. 3.30]	Choline/Glucose	1.44	6.58e-04
[3.58 .. 3.60]	Gly/Thr	1.44	9.98e-05
[3.30 .. 3.32]	Choline/Glucose	1.43	4.99e-04
[6.87 .. 6.89]	Tyr/N-acetyl-Tyr/Hydroxyphenylacetate	1.41	2.07e-03
[8.01 .. 8.03]	His	1.39	1.24e-03
[7.75 .. 7.77]	Trp	1.37	2.34e-04
[2.28 .. 2.30]	GABA/Glu	1.36	1.69e-03
[7.55 .. 7.57]	Trp	1.34	1.84e-02
[7.39 .. 7.41]	Phe	1.31	2.90e-04
[1.02 .. 1.04]	Val	1.28	3.74e-04
[8.03 .. 8.05]	His	1.26	8.47e-03

[7.99 .. 8.01]	His	1.26	2.71e-03
[3.68 .. 3.70]	Ile	1.25	4.02e-03
[4.00 .. 4.02]	Phe/His	1.23	1.88e-03
[2.26 .. 2.28]	GABA/Glu	1.22	3.23e-03
[3.60 .. 3.62]	Gly/His	1.17	1.76e-03
[2.30 .. 2.32]	GABA/Glu	1.15	3.30e-03
[7.37 .. 7.39]	Phe	1.10	3.09e-04
[4.06 .. 4.08]	Phe/His	1.08	7.46e-03
[2.64 .. 2.66]	Met	1.07	1.40e-03
[5.40 .. 5.42]	Sucrose	1.07	9.76e-05
[3.14 .. 3.16]	Phe	1.05	1.24e-03
[7.69 .. 7.71]	Trp	1.04	4.78e-04
[1.70 .. 1.72]	Agmatine/Lys/Arg	1.04	1.43e-02
[2.62 .. 2.64]	Met	1.02	4.40e-03
[2.10 .. 2.12]	Glu	1.01	2.03e-04

Table S3. Results of pathway analysis of ^{1}H NMR metabolomics data.

Pathway Name	Hits	Match Status	*p*	Impact
**19606+KHS-Cnd *vs* 19606**				
Pyruvate metabolism	Acetate; Lactate	2/23	0.0053021	0.16
Glyoxylate and dicarboxylate metabolism	Acetate; Formate	2/32	0.010198	0.00
Valine, leucine and isoleucine biosynthesis	Val	1/8	0.041231	0.00
**Ab1+KHS-Cnd *vs* Ab1**				
Glyoxylate and dicarboxylate metabolism	Acetate; Formate	2/32	0.010198	0.00
Phenylalanine, tyrosine and tryptophan biosynthesis	Phe	1/4	0.020789	0.50
Phenylalanine metabolism	Phe	1/8	0.041231	0.36
**Ab2+KHS-Cnd *vs* Ab2**				
Glyoxylate and dicarboxylate metabolism	Acetate; Formate	2/32	0.010198	0.00
Valine, leucine and isoleucine biosynthesis	Val	1/8	0.041231	0.00
**Ab4+KHS-Cnd *vs* Ab4**				
Pantothenate and CoA biosynthesis	Val; Asp	2/20	0.0059354	0.00
Glyoxylate and dicarboxylate metabolism	Acetate; Formate	2/32	0.014978	0.00
Phenylalanine, tyrosine and tryptophan biosynthesis	Phe	1/4	0.024908	0.50
Valine, leucine and isoleucine biosynthesis	Val	1/8	0.049297	0.00
Phenylalanine metabolism	Phe	1/8	0.049297	0.00
